# Botulism in Cattle: A Case Report of an Outbreak in Sardinia (Italy)

**DOI:** 10.3390/ani13152435

**Published:** 2023-07-27

**Authors:** Luigia Pinna, Annamaria Coccollone, Marcella Maxia, Luca Bano, Concetta Scalfaro, Daniela Mandas, Manuele Liciardi

**Affiliations:** 1 S.C. Complex Territorial Diagnostic Structure of Cagliari, Istituto Zooprofilattico Sperimentale of Sardinia, Via Dell’Acquedotto Romano, 09030 Elmas-Cagliari, Italy; luigia.pinna@izs-sardegna.it (L.P.); annamaria.coccollone@izs-sardegna.it (A.C.); manuel.liciardi@izs-sardegna.it (M.L.); 2 ATS Sardegna, ASSL n°7 Sulcis, 09013 Carbonia, Italy; marcella.maxia@aslsulcis.it; 3 Laboratory of Clinical Diagnostics, Istituto Zooprofilattico Sperimentale of Venezie, 35020 Legnaro, Italy; lbano@izsvenezie.it; 4 Microbiological Food Safety and Food-Borne Diseases Unit, Department of Food Safety, Nutrition and Veterinary Public Health, National Reference Centre for Botulism, Istituto Superiore di Sanità, 00161 Rome, Italy; concetta.scalfaro@iss.it

**Keywords:** cattle botulism, neurotoxins, quick diagnosis, silage

## Abstract

**Simple Summary:**

Botulism is a disease that can affect humans as well as many animals. Most cases of botulism in farm animals are foodborne. This report describes the clinical course and diagnosis of a botulism outbreak in cattle that was most likely derived from foods. We emphasize the importance of the early sampling of food and biological matrixes in cases of suspected botulism to arrive at a quick diagnosis.

**Abstract:**

*Clostridium botulinum* is the main causative agent of botulism in humans and animals. The ingestion of the botulinum neurotoxin, usually types C and D, has been shown to produce disease (neurological symptoms) in most botulism cases in cattle. We report an outbreak in Southern Sardinia that involved a livestock farm with 120 animals, 39 of which died. The aim of this report is to describe the course of this outbreak and the progression of symptoms up to the death of some animals; we also describe the therapeutic approach applied in this case and the analytical techniques used to diagnose the disease. Finally, we emphasize the importance of promptly proceeding with the sampling of several matrixes when a suspicion of botulism arises.

## 1. Introduction

Botulism is a neuromuscular disease characterized by profound generalized flaccid muscle paralysis, and it sometimes causes death. This infectious disease develops as a consequence of exposure to the neurotoxins (BoNTs) produced by bacteria of the genus Clostridium, specifically *Clostridium botulinum*. These are Gram-positive, ubiquitous, obligate anaerobic, spore-producing bacteria. They can be divided into four groups based on their ability to produce different BoNTs: group I includes the proteolytic strains that produce BoNT/A, BoNT/B, and BoNT/F; group II includes the strains that produce BoNT/E and the glucidolytic, non-proteolytic strains that produce BoNT/B and BoNT/F; group III includes the strains that produce BoNT/C, BoNT/D, and the mosaic neurotoxins C/D and D/C; and, finally, group IV contains *C. botulinum*, which produces BoNT/G, also known as *C. argentinense* [1,2,3,4,5]. Recently, a new neurotoxin (BoNT/H) was identified and characterized; sequence analyses, comparative genomics, and phylogeny established its production by strains of group I *C. botulinum* [5,6]. In addition to *C. botulinum*, some strains of *C. butyricum* and *C. baratii* are capable of producing the neurotoxins BoNT/E and BoNT/F, respectively.

Human botulism is associated with neurotoxins A, B, E, and F, and it is currently a well-managed disease; however, animal botulism represents a more serious problem that causes great economic damage due to its high mortality rate [3,7,8]. Animal pathology is mainly associated with neurotoxins C and D and their mosaic forms C/D and D/C [4,9,10,11,12].

*C. botulinum* is able to survive as a spore in several substrates for long periods, such as soil, water, and the gastrointestinal tracts of various animals. Under favorable conditions, the spores develop into vegetative forms that can produce the BoNTs to which they are genetically predisposed. These favorable conditions can be found in decaying carcasses, insufficiently acidified silage, or decaying vegetation in ponds [13]. Bovine botulism BoNTs are neurotoxins A, B, C, and D; generally, type C and D toxins are detected in substrates contaminated by the carcasses of birds or small animals (water, feed, or the environment). In Europe, the presence of the D/C serotype is predominantly observed [4,8].

In cattle, the main route of contamination is the ingestion of food, water, or carcasses containing preformed toxins; insufficiently acidified silage or silage contaminated with the carcasses of small mammals or birds; and toxins derived from poultry carcasses or small mammals present in the litter of poultry that is distributed as fertilizer or piled in pastures [7].

The first clinical signs of botulism can manifest within 24 h or after as long as two weeks, depending on the dose of BoNTs ingested by the animal. The course of the disease is variable, manifesting in peracute, acute, or chronic forms. In all forms of botulism, the most frequent symptoms are progressive paralysis with muscle weakness and tremors, while ataxia, dysphagia, a loss of tone in the tongue, mild drooling, bradycardia, decreased movement of the rumen, and recumbency have also been observed in cattle [4,14]. Death is caused by flaccid paralysis of the diaphragm, resulting in respiratory failure.

Outbreaks of bovine botulism have been reported in several parts of the world (such as areas of North America), including Europe (England and Wales, France, Turkey, and Finland). To the best of our knowledge, the first outbreak in Italy occurred in 1950, when Bianchi reported an outbreak in the province of Modena [9]. Subsequently, reports by Rosignoli in 2000, Zarenghi in 2006, and Mariano et al. in 2019 described a case of botulism that occurred in 2012 in Tuscany, with toxins D and C in the mosaic form D/C causing a high mortality rate [14,15,16]. Conversely, the episode addressed by Pirovano in 2003 included two cases of suspected botulism characterized by high mortality rates. The cases presented typical bovine botulism symptoms and disease courses but were not confirmed via laboratory analyses of samples [17].

This article describes an outbreak of bovine botulism that occurred on a farm in the Province of Medio Campidano in South-Western Sardinia (Italy) in April 2022. The symptoms were attributable to this infectious disease, which caused the deaths of 39 animals. Botulism was subsequently confirmed via diagnostic analyses.

## 2. Case Report

The livestock farm was located in a municipality in the Province of Medio Campidano, South-Western Sardinia (Italy). The site comprised two farms that represented a single epidemiological unit because the farms shared a farmer, management, equipment, and facilities. On this farm, 120 Friesian cattle were used for the production of milk and meat: 73 were adults (born by 2020), 39 were calves (born in 2021), and 8 were unweaned calves (born in 2022) (Table 1). The animals were kept in fixed stalls and fed with a feed mixture comprising corn and barley flour (70/30), soy flour, and large fibers (Martini Zootecnica) produced by the farmer himself via a mixer. Also, white hay and alfalfa hay were usually distributed once per day, early in the morning at around 7 a.m.

The feed mixture was prepared in surplus (about 100 quintals), and it lasted for about a month; this was stored in a silo. The drinking water was shared with both the farmer’s family and the other animals and came directly from the water mains without a collection tank.

### Symptoms and Clinical Course

Within 23 days, from 21 April to 13 May, 39 animals died: 37 were adults and 2 were calves (Table 1).

**Figure 1:**
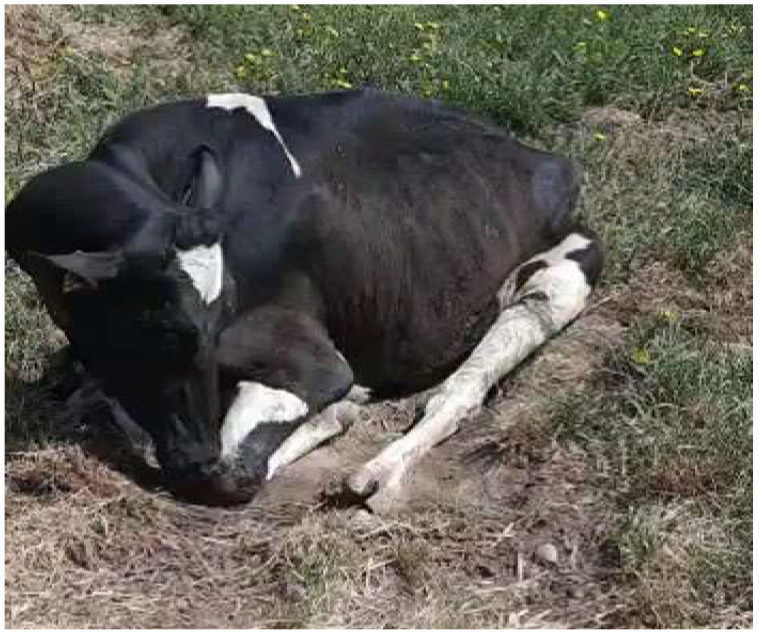
Sterno-costal recumbency of an adult cow.

**Figure 2:**
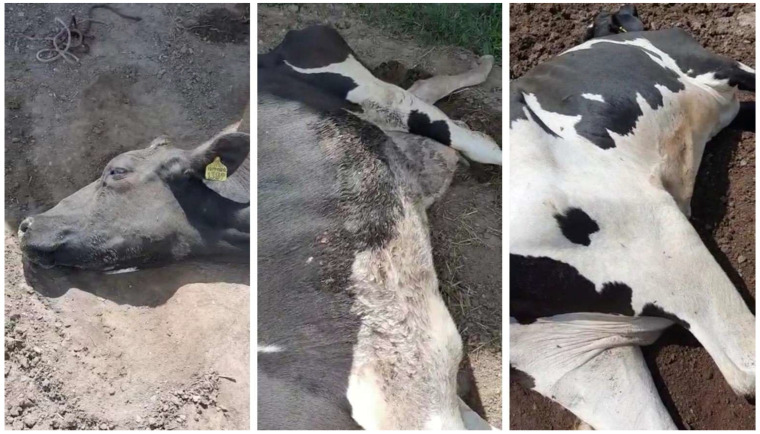
Permanent lateral recumbency.

On the first day, it the sudden death of an adult bovine without premonitory symptoms was observed, and the equally sudden sterno-costal recumbency of two other adult animals was also observed (Figure 1). Subsequently, the pathological event was characterized by an initial phase of about 24 h and a second phase lasting 48 h: in the first phase, all the symptomatic animals presented an uncertain gait, asthenia, staggering, and difficulty rising from the sterno-costal position, but somatic motor reflexes were present, with a slight atony of the tail. The state of the sensorium was maintained, as was rumination, and some animals continued to feed. In the second phase, a permanent lateral recumbency manifested itself with tetraplegia, air hunger, abdominal shallow breathing, wide-open eyes, and strabismus (Figure 2). The persistence of this state varied depending on the animal, lasting from 48 h to 5–6 days before death in all affected animals. On the 13th day, an interruption in the deaths occurred for 72 h before they resumed exclusively for the animals already in recumbency, without involving new animals. From the 16th to the 23rd day, the last three animals died (Table 2). No episodes of fever were observed among the animals involved in the outbreak.

Regarding therapy, starting on the 7th day from the beginning of the pathological event, five animals were treated with procaine benzylpenicillin, which was administered intramuscularly (Repen Fatro, 4.5–6 mL/100 kg liveweight, equal to 9000–12,000 UI of benzylpenicillin and 11.3–11.5 mg of dihydrostreptomycin/kg liveweight/die for 3 days). On 22 April, the day following the first death, a mixed clostridial vaccine (Covexin 10 Zoetis Italy S.r.l.) was administered to 75 animals, with a 30-day booster. Brewer’s yeast (200 g/head) was administered orally to all animals from the 8th day. None of the treatments resulted in a reduction in symptoms and mortality.

## 3. Materials and Methods

A macroscopic examination was performed on three adult lactating cows. Furthermore, histological examinations could only be performed for the liver, intestines and kidneys of two animals (Dead Bovines 1 and 2) because of the process of autolysis in the other organs, and the slides were subsequently observed after hematoxylin–eosin staining.

On the same organs, routine culture tests were also carried out undern both aerobic and anaerobic conditions. In particular, they were performed via the agar plate culture method, using an agar blood medium and a MacConkey agar medium. All these analyses were performed at the Istituto Zooprofilattico Sperimentale of Sardinia, Cagliari and Sassari sections.

Furthermore, hepatic and renal biochemical profiles were evaluated for seven symptomatic animals (creatinine, albumin, bilirubin, ALP, calcium, phosphorus, total protein, GGT, GOT/AST, GPT/ALT, urea, cholesterol, CPK, glucose, and triglycerides), and analyses for the main heavy metals (Cd, Cu, Pb, and Zn) were conducted via the EPA 3052 method (microwave-assisted acid digestion of siliceous and organically based matrices) and EPA 6020B method (inductively coupled plasma—mass spectrometry); mycotoxins (Aflatoxin M1) were evaluated via UPLC-MS/MS, and pesticides (organochlorines, alkaloids, organophosphates, and pyrethroids) were evaluated. Pesticides were evaluated via the QuEChERS dispersive solid-phase extraction method and an instrumental analysis was carried out via gas chromatography in the Environmental Chemistry and Toxicology Laboratory of Istituto Zooprofilattico Sperimentale of Sardinia, Sassari.

Three feces samples from different symptomatic bovines (Symptomatic Bovines 1, 2, and 3), two organs (liver and kidney) and one rumen content sample from the same dead bovine (Dead Bovine 1), and four different feed samples were sent to the Istituto Superiore di Sanità (ISS, Rome) for BONT-producing clostridia detection (Table 3). All samples were weighed (25 g), supplemented with an equal amount of phosphate-buffered gelatin, and then inoculated in 225 mL of fortified cooked meat medium (Oxoid 12.5%, soluble starch 0.1%, yeast extract 1%, calcium carbonate 0.5%, ammonium sulphate 1%, glucose 0.8%, and cysteine 0.1%). The inoculated broths were thermally treated at 70 °C for 10 min, cooled to room temperature, and incubated anaerobically at 30 °C for 96 h. After the incubation, 1 mL of each culture was subjected to DNA extraction and multiplex real-time PCR, as described by the CNRB31.012 method [18].

A further dead bovine (Dead Bovine 3) with symptoms of botulism underwent an anatomo-pathological examination; subsequently, one sample each of the liver, kidney, abomasum sample, rumen content, and intestinal contents of the same dead bovine were sent to the Treviso section of the Istituto Zooprofilattico Sperimentale of Venezie (IZS Venezie) (Table 3). Furthermore, three feces samples were also subjected to BoNT-producing clostridia detection analyses; these samples were taken from a further three symptomatic cohabiting animals (Symptomatic Bovines 4, 5, and 6; Table 3). A total of 1.5 g of each sample was added to 1.5 mL of saline and mixed in a sterile mortar and then introduced into 12 mL of fortified cooked meat medium (FCMM) [19]. The inoculated test tubes were placed in a water bath at 71 °C for 10 min and then incubated at 37 °C in an anaerobic cabinet (Shel Lab, Cornelius, OR, USA). After 48 h of incubation, 175 µL of each broth culture was collected for automatic DNA extraction (Microlab Starlet, Hamilton, Bonaduz, Switzerland) using the MagMax Total Nucleic Acid Isolation kit (Ambion/Life Technologies, Carsland, CA, USA). The extracted DNA was subjected to different PCR protocols for the detection of the genes encoding botulinum neurotoxins: one protocol for serotypes A, B, E, and F, and another protocol for serotypes C and D and their mosaics [20,21].

Furthermore, three serum samples and three rumen fluid samples from the same symptomatic animals (Symptomatic Bovines 4, 5, and 6; Table 3) were also sent to IZS Venezie (Treviso), and analyses for the detection of botulinum neurotoxins type C and D and their mosaic forms were carried out using the EndoPep-MS method. Before processing, the rumen fluid samples were supplemented with an equal volume of phosphate-buffered gelatin for 18 h at 4 °C, centrifuged for 20 min at 8000× *g*, and the supernatant was filtered using syringe filters (Millipore, Burlington, MA, USA) with a pore diameter of 0.45 µm. The obtained samples were then processed using the EndoPep-MS technique, as described in [22].

## 4. Results

**Figure 3:**
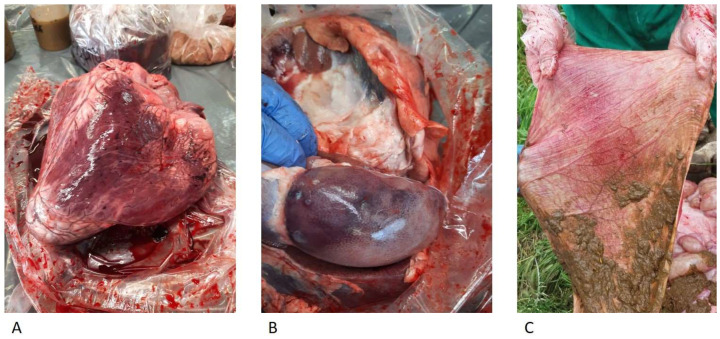
Epicardial petechial hemorrhages (**A**), hemorrhages on the renal cortex (**B**), and hemorrhagic gastroenteritis (**C**).

The anatomo-pathological examinations performed on the three animals showed hemorrhagic gastroenteritis, epicardial petechial hemorrhages, and hemorrhages on the renal cortex (Figure 3) and on visceral surface of the liver. It was possible to perform histological analyses on the organs of two animals only (Dead Bovines 1 and 2) because of the advanced autolysis process of the organs of the other animal.

The histological examination of the liver showed an alteration of the architecture of the hepatic lobule; in particular, the swelling of hepatocytes, leading to a loss of cell shape, and rare lympho-granulocytic foci in the hepatic periportal space were observed. Glomerular capillary hemorrhages were observed in the right kidney, and interstitial hemorrhages and vascular congestion were also observed. The injurious picture referred to hemorrhagic glomerulonephritis. In the other organs, the histological analysis did not allow for a diagnostic evaluation because serious and widespread autolysis phenomena were observed.

The organs affected by macroscopic lesions were subjected to routine culture tests; these allowed for the isolation of several strains of *Escherichia coli* from the livers of two animals and a strain of *Clostridium perfringens* from the kidney and mesenteric lymph node of one animal.

Analyses for the detection of heavy metals (Cd, Pb, Zn, and Cu), mycotoxins (Aflatoxin M1) and pesticides were negative. The biochemical parameters were in the normal range except for significantly increased CPK values in two animals, which were probably related to prolonged recumbency [17].

All multiplex real-time PCR analyses for the detection of BONT-producing clostridia performed at the ISS on three stool samples, one liver sample, one kidney sample, one ruminal content sample, and four feed samples yielded negative results.

Botulinum toxin D was detected in one abomasum sample, one rumen content sample from Dead Bovine 3, and in two feces samples from the Symptomatic Bovines analyzed by the Treviso section of the IZS Venezie (Table 3). Conversely, no botulinum neurotoxins of type C and D or their mosaic forms were detected by using the EndoPep-MS method on serum and rumen fluid samples analyzed by the Treviso section of the IZS Venezie.

## 5. Discussion

In cattle botulism, the main symptoms are progressive paralysis with muscle weakness and tremors, ataxia, dysphagia, a loss of tone in the tongue, mild drooling, bradycardia, decreased movement of the rumen, and recumbency; many of these symptoms are common to other forms of animal botulism. Generally, the clinical signs of botulism appear from 24 h up to 17 days, depending on the dose of BoNTs ingested by the animal [4,14,16].

The course of the episodes described in this case report could be divided into two main phases. During the first phase, the prevalent symptomatology concerned the muscles: the animals showed difficulty in gait, staggering, sterno-costal recumbency, and difficulty in standing up, while the somatic motor reflexes were maintained. In the second phase, the onset of breathing difficulties was observed, with shallow breathing in addition to recumbency, which became permanent laterally, and a state of general malaise, which lasted up to 5–6 days until death. All animals were afebrile during their clinical course.

These symptoms and the epidemiological trend (Table 2) were attributable to cases of bovine botulism, but further diagnostic investigations were performed for differential diagnosis [4,14,17].

Because of the involvement of a large number of animals, the neurologic symptoms detected, the high mortality rate, and the absence of specific anatomo-pathological and histological lesions, other diagnostic possibilities were considered. Some of these are represented by hypocalcemic collapse (milk fever), heavy metal and pesticide poisoning, or mycotoxicosis; these assumptions were subsequently excluded because of the anamnestic data, the epidemiological trend, and the analytical investigations previously described.

Intoxication by botulinum neurotoxins was later confirmed via tests of samples of the abomasum, the ruminal content derived from Dead Bovine 3, and the feces samples derived from the Symptomatic Bovines that were performed by the Laboratory of Treviso section of the IZS Venezie, using the BoNT-producing clostridia detection method [20]. In Table 2, it is possible to observe the total number of recumbent animals per day, the prevalence of recumbency, and the distribution of mortality over time; the mortality could be defined as acute and sudden during the first week and chronic in the following weeks. This trend was probably associated with the intake of contaminated feed and the quantity of BoNTs ingested by the animals, even though no traces of decomposing animal carcasses were found in the scarce residues of Unifeed stored in the silo one week after the onset of the pathological event [7].

In addition, it was difficult to establish the exact contaminated food involved and when the exposure to botulinum toxins occurred because of the variability of the incubation period of bovine botulism, which ranges from a few hours (toxin ingestion) to two weeks (ingestion of spores). Furthermore, new cases may appear a considerable length of time after the elimination of the food involved.

The negative results of the botulinum neurotoxin detection for four different foods used in breeding (hay, cornmeal, soy, and integrated feed) obtained by the Laboratory of ISS Rome could not exclude the involvement of these matrixes as a source of toxinfection due to the presence of decomposing dead animal carcasses [7]. In fact, the vegetative form of *Clostridium botulinum* could have been mixed into portions of feed within the Unifeed tank administered on previous days. We could not submit these residues to tests for the detection of *Clostridium botulinum* and its toxins.

The positive results obtained only in some samples (abomasum, rumen content, and two feces samples) suggested that in cases of suspected botulism, the timely sampling of food and biological matrixes should be carried out to avoid the degradation of neurotoxins by endogenous proteases. Therefore, the observed epidemiological trend and the negative outcome of the analyses to detect *Clostridium botulinum* and its spores in hay and silage residues indicated the contamination of a feed mixture previously administered to the animals.

## 6. Conclusions

This work demonstrates the difficulty of diagnosing this infectious disease if the diagnose is not performed quickly. Specific analytical procedures should be carried out as soon as the first symptoms attributable to botulism are observed. In this case report, the negativity detected in most samples demonstrates the importance of the rapid withdrawals of the main matrixes (such as blood in the early stages of symptoms, food, feces, ruminal contents, and organs if there are dead animals) so as to increase the chances of isolating the clostridia or at least detecting toxins.

No therapy was curative for the episode studied herein; in fact, following the antibiotic therapy and clostridium vaccine administered to the farm animals, none of the treatments resulted in a reduction in symptoms and mortality. In general, any therapy should not be administered before obtaining a certain diagnosis because of the risk of aggravating an uncertain pathological situation. Furthermore, antibiotic therapy is not recommended in cases of botulism because of the possible release of BoNT following cell lysis [23].

This infectious disease is currently underestimated, especially when episodes involve few animals. Isolating BoNT-producing clostridia is difficult, even in cases of animals with overt symptoms [17].

To conclude, to our knowledge, this represents the first report of botulism in Sardinian cattle, which we hope will act as a stimulus for the development of rapid control methods for farms in order to more effectively manage such episodes of considerable mortality.

## Figures and Tables

**Table 1:** Number of total animals and dead animals on farm 1 (**A**) and farm 2 (**B**).

**A**				
	Adults	Calves	Unweaned Calves	Total Animals
FARM 1	54	34	5	93
DEAD ANIMALS	28	2	0	30
**B**				
	Adults	Calves	Unweaned Calves	Total Animals
FARM 2	19	5	3	27
DEAD ANIMALS	9	0	0	9

**Table 2:** Course of the pathological episode.

Farm Inspection	N° Dead Animals	N° New Recumbent Animals Per Day	N° Total Recumbent Animals Per Day	Prevalence of Recumbent Animals (%)
**Day 1 (21 April)**	1	2	2	1.7
**Day 2 (22 April)**	3	2	4	3.4
**Day 3 (24 April)**	6	4	5	4.5
**Day 4 (25 April)**	2	3	7	6.5
**Day 5 (26 April)**	7	3	7	6.9
**Day 6 (27 April)**	1	1	7	7
**Day 7 (28 April)**	4	2	8	8.3
**Day 8 (29 April)**	4	-	8	8.7
**Day 9 (30 April)**	1	3	9	9.9
**Day 10 (2 May)**	4	-	6	6.9
**Day 11 (3 May)**	1	-	6	7.0
**Day 12 (5 May)**	2	-	6	7.1
**Day 13 (6 May)**	0	-	4	4.8
**Day 14 (7 May)**	0	-	4	4.8
**Day 15 (8 May)**	0	-	4	4.8
**Day 16 (9 May)**	1	1	5	6
**Day 17–23 (12–18 May)**	2	-	2	2.5

**Table 3:** Analyzed samples and the laboratories that performed the histological and molecular analyses.

	IZS Sardinia	ISS Rome	IZS Venezie
Dead Bovine 1	Liver, intestine, and kidney	Liver, kidney, and rumen contents	-
Dead Bovine 2	Liver, intestine, and kidney	-	-
Dead Bovine 3	-	-	Liver, kidney, abomasum sample, rumen content, and intestinal content
Symptomatic Bovine 1	-	Feces	-
Symptomatic Bovine 2	-	Feces	-
Symptomatic Bovine 3	-	Feces	-
Symptomatic Bovine 4	-	-	Feces, serum, and rumen fluid
Symptomatic Bovine 5	-	-	Feces, serum, and rumen fluid
Symptomatic Bovine 6	-	-	Feces, serum, and rumen fluid
-	-	Feed (4 different samples)	-

## Data Availability

Data sharing is not applicable to this article.

## References

[1] Rasetti-Escargueil C., Popoff M.R. (2019). Antibodies and Vaccines against Botulinum Toxins: Available Measures and Novel Approaches. Toxins.

[2] Peck M.W., Smith T.J., Anniballi F., Austin J.W., Bano L., Bradshaw M., Stringer S.C. (2017). Historical perspectives and guidelines for botulinum neurotoxin subtype nomenclature. Toxins.

[3] Skarin H., Tevell Åberg A., Woudstra C., Hansen T., Löfström C., Koene M., Bano L., Hedeland M., Anniballi F., De Medici D. (2013). The workshop on animal botulism in Europe. Biosecur. Bioterror..

[4] Anniballi F., Fiore A., Löfström C., Skarin H., Auricchio B., Woudstra C., Bano L., Segerman B., Koene M., Båverud V. (2013). Management of animal botulism outbreaks: From clinical suspicion to practical countermeasures to prevent or minimize outbreaks. Biosecur. Bioterror..

[5] Rossetto O., Pirazzini M., Montecucco C. (2014). *Botulinum neurotoxins*: Genetic, structural and mechanistic insights. Nat. Rev. Microbiol..

[6] Dover N., Barash J.R., Hill K.K., Xie G., Arnon S.S. (2013). Molecular characterization of a novel botulinum neurotoxin type H gene. J. Infect. Dis..

[7] Relun A., Dorso L., Douart A., Chartier C., Guatteo R., Mazuet C., Popoff M.R., Assié S. (2017). A large outbreak of bovine botulism possibly linked to a massive contamination of grass silage by type D/C *Clostridium botulinum* spores on a farm with dairy and poultry operations. Epidemiol. Infect..

[8] Le Maréchal C., Hulin O., Macé S., Chuzeville C., Rouxel S., Poëzevara T., Mazuet C., Pozet F., Sellal E., Martin L. (2019). A Case Report of a Botulism Outbreak in Beef Cattle Due to the Contamination of Wheat by a Roaming Cat Carcass: From the Suspicion to the Management of the Outbreak. Animals.

[9] Bianchi E. (1950). Botulism in cattle in underestimated. Clin. Vet..

[10] Haagsma J., Ter Laak E. (1979). First case of type D botulism in cattle in the Netherlands. Tijdschr. Diergeneeskd..

[11] Jean D., Fecteau G., Scott D., Higgins R., Quessy S. (1995). *Clostridium botulinum* type C intoxication in feedlot steers being fed ensiled poultry litter. Can. Vet. J..

[12] Fillo S., Giordani F., Tonon E., Drigo I., Anselmo A., Fortunato A., Bano L. (2021). Extensive Genome Exploration of *Clostridium botulinum* Group III Field Strains. Microorganisms.

[13] Deprez P.R., Mainil J. (2006). Tetanus and botulism in animals. Clostridia in Medical, Veterinary and Food Microbiology—Diagnosis and Typing.

[14] Mariano V., Nardi A., Gradassi S., De Santis P., Anniballi F., Bilei S., Scholl F., Auricchio B., Bielli C., Culicchi M. (2019). A severe outbreak of botulism in cattle in Central Italy. Vet. Ital..

[15] Rosignoli C., Nigrelli A., Maffezzoli G., Consadori G., Costa A., Sposetti G. (2000). Episodio di botulismo in un allevamento di bovini da latte. Atti Soc. Ital. Buiat..

[16] Zarenghi L., Barigazzi G., Rastelli G. (2006). A high fatality rate botulism outbreak in a dairy heifers herd. Buiatria.

[17] Pirovano A., Luini M., Vezzoli F., Bovera C., Gualdi V. (2003). Casi di sospetto botulismo in un allevamento di bovini da carne. Large Anim. Rev..

[18] Method CNRB31.012. https://www.iss.it/documents/20126/0/Metodo_CNRB31.012.pdf/aab30bfc-794f-ada0-abab-0ab9b702eb33?t=1615188484372.

[19] Kurazono H., Shimozawa K., Sakaguchi G., Takahashi M., Shimizu T., Kondo H. (1985). Botulism among penned pheasants and protection by vaccination with C1 toxoid. Res. Vet. Sci..

[20] De Medici D., Anniballi F., Wyatt G.M., Lindstrom M., Messelhäusser U., Aldus C.F., Delibato E., Korkeala H., Peck M.W., Fenicia L. (2009). Multiplex PCR for detection of botulinum neurotoxin-producing clostridia in clinical, food, and environmental samples. Appl. Environ. Microbiol..

[21] Woudstra C., Skarin H., Anniballi F., Fenicia L., Bano L., Drigo I., Koene M., Bäyon-Auboyer M.H., Buffereau J.P., De Medici D. (2012). Neurotoxin gene profiling of *Clostridium botulinum* types C and D native to different countries within Europe. Appl. Environ. Microbiol..

[22] Drigo I., Tonon E., Pascoletti S., Anniballi F., Kalb S.R., Bano L. (2020). Detection of active BoNT/C and D by EndoPep-MS using MALDI biotyper instrument and comparison with the mouse test bioassay. Toxins.

[23] Jeffery I.A., Karim S. (2022). Botulism. StatPearls.

